# Nanoionics and Nanocatalysts: Conformal Mesoporous Surface Scaffold for Cathode of Solid Oxide Fuel Cells

**DOI:** 10.1038/srep32997

**Published:** 2016-09-08

**Authors:** Yun Chen, Kirk Gerdes, Xueyan Song

**Affiliations:** 1 Department of Mechanical & Aerospace Engineering, West Virginia University, Morgantown, WV 26506, USA; 2 U.S. DOE, National Energy Technology Laboratory, Morgantown, WV 26507, USA; 3 Department of Mechanical & Aerospace Engineering, West Virginia University, Morgantown, WV 26506, USA

## Abstract

Nanoionics has become increasingly important in devices and systems related to energy conversion and storage. Nevertheless, nanoionics and nanostructured electrodes development has been challenging for solid oxide fuel cells (SOFCs) owing to many reasons including poor stability of the nanocrystals during fabrication of SOFCs at elevated temperatures. In this study, a conformal mesoporous ZrO_2_ nanoionic network was formed on the surface of La_1−x_Sr_x_MnO_3_/yttria-stabilized zirconia (LSM/YSZ) cathode backbone using Atomic Layer Deposition (ALD) and thermal treatment. The surface layer nanoionic network possesses open mesopores for gas penetration, and features a high density of grain boundaries for enhanced ion-transport. The mesoporous nanoionic network is remarkably stable and retains the same morphology after electrochemical operation at high temperatures of 650–800 °C for 400 hours. The stable mesoporous ZrO_2_ nanoionic network is further utilized to anchor catalytic Pt nanocrystals and create a nanocomposite that is stable at elevated temperatures. The power density of the ALD modified and inherently functional commercial cells exhibited enhancement by a factor of 1.5–1.7 operated at 0.8 V at 750 °C.

The promise of nanoionics (nanocrystalline ionic materials) has significantly stimulated and advanced the research and development of novel devices for energy conversion and storage including rechargeable batteries^1^,^2^,^3^, owing to enhanced ionic transport compared to conventional materials. Nanoionics hold greater promise for Solid Oxide Fuel Cells (SOFCs) that incorporate oxygen ion conducting ceramics in the electrolyte and composite electrodes. Compared to other fuel cell varieties^4^, SOFCs offer higher chemical to electrical energy conversion efficiency and utilize various fuels including hydrocarbons derived from natural gas^5^,^6^,^7^,^8^,^9^,^10^. For oxygen ion conductors including doped zirconia and ceria that are commonly employed in SOFCs, enormous efforts are underway to enhance conductivity by manipulation of interfaces in the nanocrystalline materials^11^,^12^. Although the reported data on the conductivity of different nanostructured systems remain disjointed, compelling theoretical and experimental evidence indicates that ionic conductivity, at high temperatures that SOFCs operate, could be significantly improved owing to the fast ion transport along grain boundaries and interfaces^13^,^14^,^15^,^16^,^17^. Application of nanoionics with enhanced conductivity will allow SOFC stacks that normally operate from 650 °C to 800 °C to operate at the lower end of this range, and subsequently mitigate degradation, reduce sealing problems, enable utilization of less expensive materials, and improve response to rapid start-up and repeat thermal cycling from ambient to operating temperatures^18^.

Regardless of tremendous efforts^2^,^19^,^20^,^21^,^22^, the development of nanoionics for practical SOFCs has been extremely challenging during the past three decades owing to three major obstacles. Firstly, the structural stability of the nanostructured materials is thermally sensitive at even modest temperature of ~400 °C 23,24 because of the large surface-to-volume ratio and high surface energy of nanocrystals. Secondly, nanoionics with increased conductivity including multilayer systems^25^,^26^,^27^ are usually developed using thin film deposition techniques such as pulsed laser deposition (PLD) for flat single or polycrystal substrates. When applied in the porous SOFC electrode, the thin film deposition technique must be altered to produce conformal and uniform layer/multilayers within the porous active layer of the electrodes, typically at least 50 μm below the terminal electrode surface. Lastly, as the nanoionics are applied to the SOFC composite electrode, the nanoionics layer must be mesoporous for gas penetration and subsequent electrochemical reaction at the triple phase boundaries (TPBs). Mesoporous metal oxide frameworks for high temperature applications have been very challenging to develop during the past decade, since heat treating the materials at high temperatures normally results in morphology changes and loss of mesoporous structure^28^. For oxide ionic conductors, it is worthwhile to note that the effective single/multi layered nanoionics with enhanced conductivity developed so far for doped zirconia and ceria are normally dense films lacking pores. Consequently, the research and application of mesoporous nanoionics for SOFC remain immature and no reports are available on mesoporous nanoionics developed for inherently functional commercial SOFCs.

Here we demonstrate, for the first time, the establishment of conformal mesoporous nanoionics ZrO_2_ networks on the interior surface of porous commercial SOFC cathode formed through atomic layer deposition (ALD)^29^,^30^,^31^,^32^,^33^. By contrast to the PLD thin film deposition and chemical solution based infiltration^34^,^35^,^36^, chemical vapor based ALD is uniquely suitable for depositing uniform and conformal films on SOFC cathodes possessing complex three-dimensional topographies with high aspect ratio. Facilitated by the mesoporous nanoionic network created on the cathode interior surface, the cell exhibited much enhanced power density by a factor of 1.5, accompanied by simultaneously lowered series and polarization resistance. The network is remarkably stable and retained the same nano morphology and the mesopores after operation at 650–800 °C for 400 hours. Furthermore, the stable and conformal mesoporous ZrO_2_ nanoionic network is utilized to anchor catalytic Pt nanocrystals, prevent the agglomeration of the catalytic nanocrystals at elevated temperatures, and stabilize an engineered nanocomposite. By forming the three dimensional surface architecture consisting of both mesoporous nanoionics and nanocatalyst on the LSM/YSZ host cathode, a large overall power density enhancement at 750 °C was achieved from inherently functional SOFCs by a factor of 1.7, significantly higher than performance enhancement factor of ~1.3 achieved using solution based infiltration^34^,^35^,^36^.

## Results and Discussion

**Figure 1:**
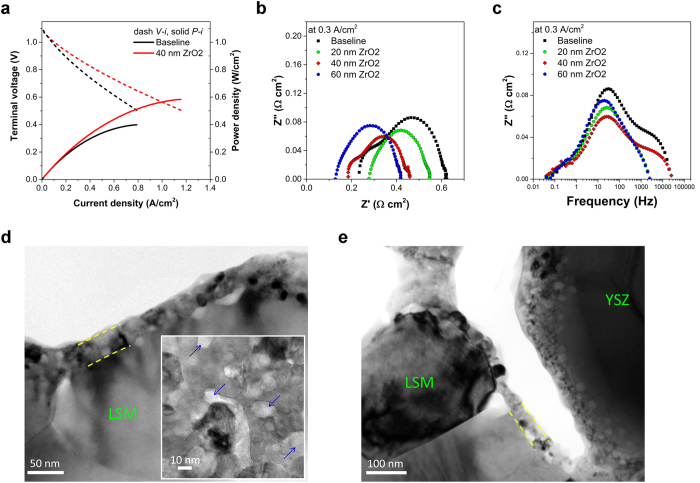
Cell performance upon operation at 750 °C and nanostructure. (**a)** Performance of the baseline cell (cell #1) and cell #3 with 40 nm ZrO_2_. (**b**) Nyquist plot of cell #1 (the baseline cell, in red squares), cell #2 (20 nm ZrO_2_, in green circles), cell #3 (40 nm ZrO_2_, in magenta diamonds) and cell #4 (60 nm ZrO_2_, in blue pentagons), showing the significant decrease of *R*_s_ and *R*_total_ of the coated cells except *R*_s_ of cell #2. (**c**) Bode plot of cell #1, cell #2, cell #3 and cell #4 showing the trend of the intermediate-frequency arc in the range of 1–100 Hz. (**d**) Cross section TEM image shows the ~40 nm porous nano ZrO_2_ layer on the LSM/YSZ backbone. The insert plan-view image shows that the ZrO_2_ surface layer is nano-grained and porous. The blue-arrowed areas are mesopores randomly distributed through the entire ZrO_2_ surface layer. (**e**), TEM image shows no significant change of ALD ZrO_2_ surface layer after 400 hours operation.

In the present work, a total of seven cells with six of the cells having differently engineered surface architectures on the cathode were investigated. All the cells and their corresponding performances are listed in Table 1. For three cells (#2, #3 and #4), a pure ZrO_x_ layer was deposited by ALD onto the LSM/YSZ cathode backbone of commercial anode-supported SOFCs. The as-deposited state of the ZrO_x_ layer is amorphous and conformal on the cathode backbone. By controlling the ALD processing cycles, a uniform layer with thickness of 20, 40 and 60 nm was deposited on the cathode of three cells, respectively. All cells were subjected to heat treatment to crystallize the ZrO_2_ structure before cell operation. Subsequently, an electrochemical test was performed at 750 °C in H_2_ and air for anode and cathode, respectively. In comparison with the baseline cell (cell #1), power density increases were observed for all three cells (in Table 1). In particular, as shown in Fig. 1a–c, a large power density increase by a factor of 1.5, accompanied by the simultaneous large decrease of both series resistance *R*_s_ and polarization resistance *R*_p_ was observed for cell #3 with 40 nm ZrO_2_ coating layer.

The TEM image of Fig. 1d reveals that a ~40 nm thick porous ZrO_2_ surface layer developed in cell #3. The polycrystalline ZrO_2_ layer possesses the nanosized ZrO_2_ grains of 20–30 nm, and features mesopores (with ~15 nm in dimension and volume fraction of ~10%) randomly distributed among the nanograins. Similar grain sizes in the range of 20–30 nm are observed in cell #2 (20 nm thick initial coating layer) and cell #4 (60 nm thick initial coating layer) as well. Under the same ALD processing and heat-treatment conditions, the surface layer thickness has little effect on resultant surface layer grain size, or on the size and density of the mesopores. Dopants (such as Y) are not added in the ZrO_2_ ALD layer, yet the ZrO_2_ nanograins possess tetragonal crystal structure (as evidenced in Supplementary Figure 1 the high resolution TEM image and the corresponding Fast Fourier Transformation). The observation is consistent with literature reports indicating that in nanocrystalline undoped zirconia the tetragonal phase that is normally stable above 1400 K, could be stabilized at room temperature in the ALD films^37^,^38^. Approximately 3.5 at% Mn (calculated as Mn/(Mn + Zr)) is detected in the ZrO_2_ nano-layer under TEM using Energy Dispersive Spectroscopy (EDS) analysis. Such Mn incorporation, presumably diffused from the neighboring LSM grains, could play favorable roles promoting ionic conduction and electron transfer in ZrO_2_ layer^39^.

The impedance data of cells #2, #3, and #4 along with #1 are compared in Fig. 1b,c. For all three cells of #2, #3, and #4, the series resistance *R*_s_ systematically decreased as the initial surface layer thickness increased from 20 nm to 60 nm, as indicated in Table 1. The *R*_s_ of cell #4 with 60 nm layer thicknesses is about 50% of that from the baseline cell #1. On the other hand, the thickness of ALD layer also greatly influenced the polarization resistance *R*_p_ of the cells. *R*_p_ of all three ZrO_2_ coated cells is lower compared to the baseline cell, and *R*_p_ is lowest for cell #3 with 40 nm thick layer. However, *R*_p_ of cell #4 with 60 nm thick layer is the highest.

For the systematic change of *R*_s_ and *R*_p_ as the function of the initial ALD layer thickness, further consideration is necessary to understand the mechanisms by which an ostensibly electronic insulator (ZrO_2_) placed on the electrode surface and yet facilitate the oxygen reduction reaction, a process which necessarily involves an electron transfer. As stated earlier, the ALD layer is having tetragonal structure with Mn incorporation. It is possible that, through the course of fabrication and further operation, the ZrO_2_ nanoparticles became doped with Mn (via cation diffusion) and become a mixed conductor. Certainly such a phase would increase TPB length (therefore decreasing *R*_p_)^40^, but it is not clear that the ohmic resistance measured in the cell would decrease unless the overall conductivity (both the surface and bulk conductivity) of electrode was improved. Given that the LSM is already an adequate electronic conductor, it seems unlikely that improved conductivity will arise in the bulk electrode, and indeed enhanced conductivity is not customarily attributed to an applied electrocatalyst or infiltrate on the electrode surface, as indicated in the cited references^34^. On the other hand, it is certainly plausible given published research that the physical structures of ZrO_2_ layer on the electrode surface created and examined here will exhibit enhanced ionic conductivity, and could manifest as a decrease in the measured ohmic resistance if the global ionic transport resistance is appreciably decreased. In this study, the ZrO_2_ layer of three cells featuring different thicknesses share the same mesoporous structure on the LSM backbone, accordingly, conduction along the ZrO_2_/LSM interface should be constant for all three samples. Since the three cells with different thicknesses also share the same ZrO_2_ nanograin size, the observation of decreased *R*_s_ with the increase of layer thickness clearly implies that the high density of grain boundaries, that increased as the layer thickness increases, between neighboring ZrO_2_ nanograins within in ALD surface layer could correlate to faster ion transport at 750 °C.

The change of the *R*_p_ with the layer thickness can also be explained in terms of the connectivity of the nanograins and the percolation of the mesoporous network within the ZrO_2_ surface layer. It is expected that if nanoionics transport is an active mechanism, the applied ZrO_2_ structures will increase the total length of the TPB (e.g. when ZrO_2_ deposits on LSM), and will manifest as the observed decrease in polarization resistance. The cell possessing the 40 nm coating features *R*_p_ lower than that of the cell with a 20 nm coating, possibly owing to enhanced connectivity for ionic conducting ZrO_2_ grains on the LSM surface, because ALD layers of the different cells share the same grain size of 20–30 nm and the pore size of ~15 nm. Even though the total TPB density is high for the cell with 20 nm thick coating, the local connectivity between the ZrO_2_ grains on LSM surfaces may be diminished relative to the 40 nm layer. Consequently, the effective TPB density^41^,^42^ may actually be smaller for the cathode with 20 nm coating than that for cell with 40 nm coating. On the other hand, when the layer thickness is increased to 60 nm, which is ~2–3 times thicker than the grain dimension and ~4 times of the mesopore size, the effective TPB density may actually be lower owing to the loss of the open mesopores for gas penetration. As a result, higher *R*_p_ is observed for the 60 nm ZrO_2_ layer than that from the cells with 20 and 40 nm layer thicknesses.

Overall, the 40 nm coating layer produces the highest power density presumably due to the compromise between the density of effective TPBs and the density of ZrO_2_/ZrO_2_ grain boundaries. The present data demonstrate the importance of grain boundaries and open mesopores in the ALD layer to ionic transport and associated electrochemical reactions. Other considerations and indeed further experiments are required to completely explain the fundamental source of improved performance in the engineered system, and although complete research is reserved for a subsequent effort, possibilities of additional nanoionic transport through ALD coating layer is worthwhile to be pointed out here.

**Figure 2:**
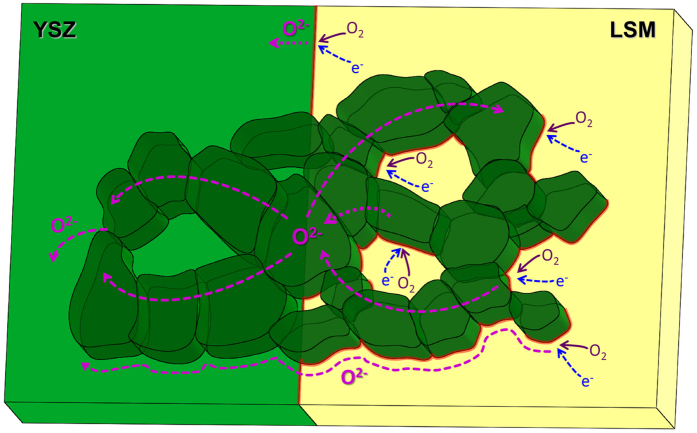
A schematic image showing the nanoionic network with the path of transport for electrons and oxide ions for the LSM/YSZ with ZrO_2_ mesoporous network. Red line illustrates the active sites (TPBs) for the oxygen reduction reaction; and the pink dashed lines illustrate the paths for ionic transport.

With the consideration of the ionic transport at the ALD surface layer, and the increased effective TPBs, the morphology of the coating layer and the local ion transport for the 40 nm ZrO_2_ nanoionic network are depicted in Fig. 2. Near the mesopores, the interface between the ZrO_2_ and LSM from the coating layer acts as additional effective TPBs. Within the thin-layered nanoionic network on the LSM backbone, the ZrO_2_/ZrO_2_ grain boundary planes were exposed to the gas, and promote fast ion transport. Resulting from the open mesopores, the good connectivity between the neighboring grains, as well as high density of the surface grain boundaries and interfaces, the nanoionic network makes the entire cathode surface become ionically conducting and electrochemically active. Most importantly, the surface nanoionic network on LSM provides local ionic transport pathways, thus the overall oxygen surface exchange and transport kinetics on the cathode surface were significantly improved.

Remarkably, the nanoionic network exhibits high stability upon electrochemical operation under high temperature (650–800 °C), and in the presence of representative industry operation relevant electrical current densities, and potential gradients. After cell operation for 400 hours (from cell #3), the nano-grained ZrO_2_ coating depicted in Fig. 1e retains the as-fabricated porosity and grain size.

The stable ALD layer could also be utilized to pin the nano-catalyst because of the porosity within the surface layer. The mesoporous ZrO_2_ nanoionic network is thus subsequently engineered to generate a heterostructured surface scaffold that anchors nanocatalysts and further improves the cell performance. Transition metal catalysts (e.g. nanosized Pt) are considered excellent catalysts for oxygen reaction reduction enhancement^34^,^43^,^44^, to increase the cathode performance. However, elevated temperatures stimulate grain growth of nanocrystals, and cause the reduction of active surface area and catalytic activity. In the present study, by using ALD technique, a conformal Pt nanocatalyst layer was deposited onto the internal surface of LSM/YSZ backbone. The as-deposited Pt particles are crystalline and discrete, possessing ~3 nm in diameter, and a total layer thickness ~7 nm (in Supplementary Figure 2a (from cell #5)). The Pt loading is estimated to be minute at only ~1.5 × 10^−3^ mg/cm^2^. The Pt-deposited cell (cell #5) was operated at 750 °C and a performance enhancement factor of 1.4 was achieved (Table 1). Microscopy study indicates as-fabricated ~3 nm Pt catalyst particles agglomerate to ~100 nm after operation (in Supplementary Figure 2b), and lose the active surface for reactions. The diminished effectiveness of the Pt nano-particles and concomitant loss of the activation site abundance is intuitively predictable. Nevertheless, an obvious cell power density enhancement was achieved, accompanied by a significant reduction in *R*_p_, but no obvious changes in *R*_s_ (Table 1). These results implied the opportunity to further improve the cell performance by stabilizing the Pt nano particles.

**Figure 3:**
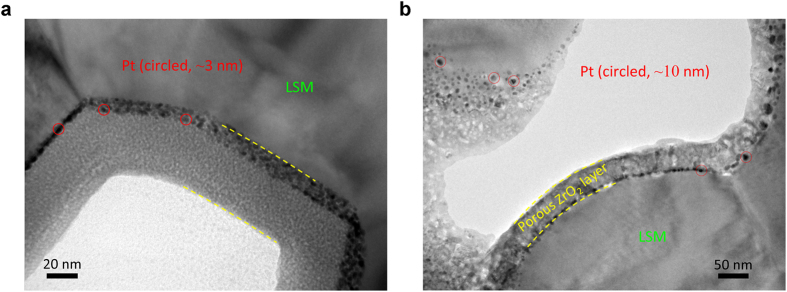
A two-phase coating of superjacent ZrO_2_ layer subjacent Pt (cell #6). (**a**) As-deposited state of the ~3 nm Pt layer and the 40 nm amorphous ZrO_x_ layer. (**b**) Conformal amorphous ZrO_x_ layer turned into a continuous crystalline ZrO_2_ layer covering the entire backbone of the cathode.

The unique architectural and crystalline character of each as-deposited ALD layer (amorphous ZrO_x_ or crystalline Pt) offers an opportunity to engineer architecture by leveraging the thermodynamically controlled characteristics of the constituent phases. Specially, the strategy is to pin Pt particles to the cathode surface using stable ZrO_2_ nanoionic network, therefore to retain the highly active electrocatalytic structure even when the system is subjected to aggressive driving potentials at elevated temperatures. One approach successfully applied a two-phase coating of amorphous ZrO_x_ on a backbone-deposited Pt layer composed of discrete ~3 nm Pt crystallites. As shown in Fig. 3a (from cell #6) the ZrO_x_ coating layer is ~40 nm thick, and pins the Pt particles to the backbone surface. The as-deposited ZrO_x_ is amorphous and homogeneous, therefore the nanovoids (~1–3 nm in dimension, as shown in Supplementary Figure 2a) between neighboring Pt crystals were filled with amorphous ZrO_x_. This layered structure, with superjacent amorphous ZrO_x_ and subjacent crystalline Pt, was subjected to heat-treatment before cell operation. As shown in Fig. 3b (and enlarged view in Supplementary Figure 3), the amorphous ZrO_x_ conformal layer transformed into a crystalline ZrO_2_ layer covering the entire backbone of the cathode. The layered ZrO_2_ architecture contains mesopores that preserve the gas pathway and disrupt agglomeration of the discrete Pt particles of ~10 nm that were fully pinned to the backbone surface. Remarkably, the engineered architecture depicted in Fig. 3b results in a performance enhancement of commercial button cells by a factor of 1.6 at 0.8 V (in Table 1).

**Figure 4:**
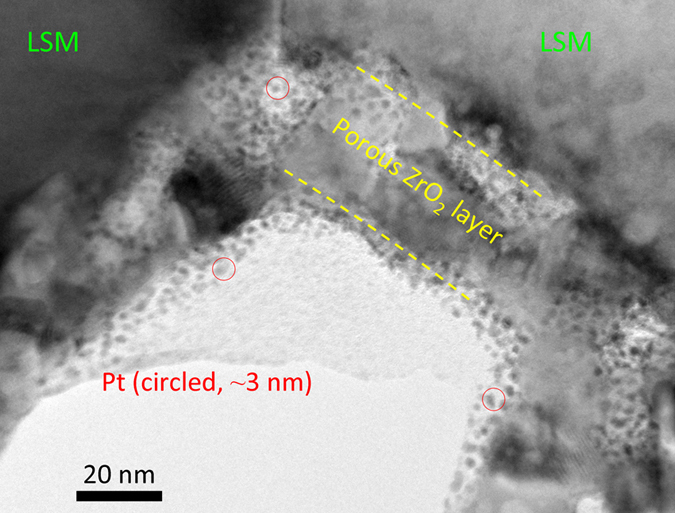
As-deposited state of a two-phase coating of ZrO_2_ layer with Pt nano-catalyst infiltrated into the meso-porous region of the ZrO_2_ layer (cell #7).

**Figure 5:**
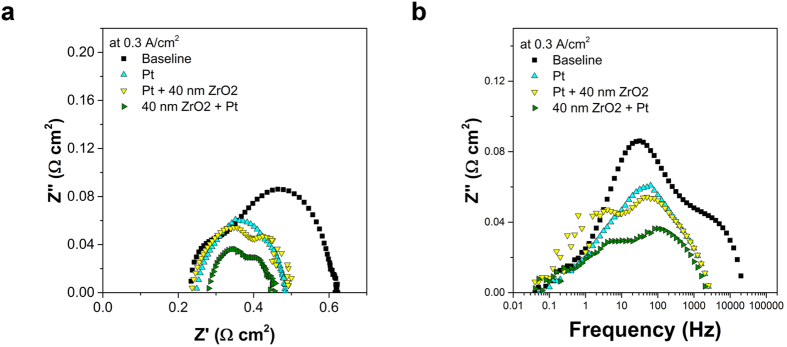
Electrochemical impedance spectroscopy of cells at 750 °C. (**a**) Nyquist plot of cell #1 (the baseline cell, in black squares), cell #5 (~10 nm Pt, in cyan triangles), cell #6 (Pt + 40 nm ZrO_2_, in yellow triangles) and cell #7 (40 nm ZrO_2_ + Pt in olive triangles). (**b**) Bode plot of cell #1, cell #5, cell #6 and cell #7 showing the trend of the intermediate-frequency arc in the range of 1–100 Hz.

An alternative engineered structure is created by applying Pt nano-particles into the mesopore region of a ZrO_2_ conformal surface layer using ALD processing. In this approach, cell #7 with 40 nm coated ZrO_x_ layers were subjected to one thermal treatment and subsequent ALD processing for Pt deposition. Figure 4 (from cell #7) depicts a subjacent ZrO_2_ coating layer 40 nm thick in which the original ZrO_2_ mesopore regions were decorated by ~3 nm Pt crystallites. After thermal treatment, the Pt particles possess diameter of ~10 nm, and disperse on the ZrO_2_ surface and inside the original pore regions (depicted in Supplementary Figure 4). Such surface structural engineering prevents evolution of Pt particle diameter from 10 nm to 100 nm, and preserves electro-catalytic activity. For commercial inherently functional full cell, a large performance enhancement factor of 1.7, significantly higher than performance enhancement factor of ~1.3 achieved using solution based infiltration^34^,^35^,^36^, is also listed in Table 1 and clearly illustrates the realizable advantages. To understand such large performance enhancement induced by various surface scaffold, it is critical to consider the evidence of the impedance analyses that were completed for each cell after operation at approximately 48 hours as seen in Figs 1b,c and 5a,b, and Table 1. Two peaks are discernable in all of the impedance spectroscopy traces derived from samples containing ZrO_2_, though the exhibited peak frequencies are broadly distinguishable based on the bulk structure. For the baseline cell and cells with only ZrO_2_, the two peaks are convoluted and the peak frequency of the larger arc is approximately 1 × 10^1^ Hz at 0.3 A/cm^2^. When Pt is introduced alone, the peak frequency shifts to 3 × 10^1^ Hz. When Pt and ZrO_2_ are both introduced, the peaks separate, equalize in magnitude, and separate to 2 to 4 × 10^0^ Hz and 5 to 10 × 10^1^ Hz. As discussed earlier, tetragonal ZrO_2_ on LSM would increase triple phase boundary length (therefore decreasing *R*_p_). Nevertheless, an increase in triple phase boundary length will not result in significant shifting of the impedance peak frequency unless the mechanism of transport has also been altered. It is possible that introduction of the ZrO_2_ + Pt phase has both the effect of increasing TPB length and influencing the global reaction mechanism, which does not preclude the introduction of new ionic transport pathways through the ZrO_2_ crystallite interfaces.

Regardless of the fundamental basis for the observed performance improvement following surface engineering via ALD, it is apparent that the structure is stable and engenders a performance increase that is atypical in magnitude and evidence indicates a potentially novel reaction mechanism. More fundamental examinations that endeavor to affirmatively correlate structure, function, and observable performance are reserved for the subsequent research effort.

### Summary

The present work demonstrated the formation of the conformal mesoporous nanoionics on the cathode surface and its impact to the performance of the inherently functional SOFC. The power density enhancement and the reduction of the reaction resistances is facilitated by the conductivity of nanoionics and simultaneous increase of effective TPB density on the cathode backbone. Remarkably, the nanoionic network is stable for electrochemical operations lasting over 400 hours, under commercially relevant conditions of temperatures (650–800 °C), and in the presence of representative electrical current densities, and potential gradients. By forming a nanocomposite with minute Pt nano-catalyst loading into the mesoporous nanoionics to facilitate oxygen reduction reaction activity, the observed performance enhancement is attributed to high surface area and associated high catalytic activity. Nanoionics and nanostructured electrode, which are extraordinary important for ion transport in the other electrochemical systems, have been very challenging to develop for SOFCs. To the authors’ knowledge, the present work is the first demonstration of establishment of nanoionic network for applications of high temperature SOFCs. These experiments contribute to validation of the nanoionic mechanisms reported previously by independent researchers and offer justification for tailoring surface structure of the electrode, via nanostructural engineering with ALD and thermo-treatment. The cell performance could be further improved by tuning the dopant distribution, the thickness of the nanoionics, as well as by adjusting the species, size, and the distribution of the catalysts. In addition to the SOFCs, the stable mesoporous nanoionic network and nanocatalyst presented in this work also opens further research for their potential applications in catalysts, photocatalyst, sensors and electrode materials, especially for those subjected to high temperatures in harsh environments.

## Experimental Section

Commercially available, anode supported solid oxide button cells fabricated by Materials and Systems Research, Inc. (MSRI, Salt Lake City, UT) were employed for all the experiments described in this paper. MSRI cells are composed of five layers as follows, starting from the anode: ~0.9 mm thick Ni/YSZ cermet layer which supports the cell structure; 15 μm thick Ni/YSZ active layer; ~12 μm thick YSZ electrolyte; ~15 μm thick La_0.8_Sr_0.2_MnO_3_ (LSM)/YSZ active layer; and 50 μm thick, pure LSM current collecting layer. The cell active area (limited by the cathode) is 2 cm^2^. The exposure area of the anode to fuel is about 3.5 cm^2^. Total 7 cells from the same batch of the commercial cells were subsequently processed and studied.

All ALD processes were performed on a Savannah 200 Atomic Layer Deposition system. The tetrakis(dimethylamino)zirconium(IV), (99% Strem Chemicals, Inc), the (trimethyl)methylcyclopentadienylplatinum(IV), (99%, Strem Chemicals, Inc) and deionized water were used as Zr precursor, Pt precursor and oxidant, respectively. During the processing of growing a ZrO_x_ amorphous layer, the sample stage was firstly pre-heated to 260 °C, and then total 35 (or 70) ALD cycles were performed to build up a 20 (or 40) nm layer. The ZrO_x_ layer was subsequently heat-treated at 850 °C for crystallization before cell operation. Similarly, for growing a Pt layer or infiltrating Pt particles, a processing with total 100 ALD cycles was performed on a pre-heated sample stage (310 °C).

SOFC button cells were tested on a test stand. Gold mesh/nickel paste and platinum mesh/platinum paste were used for anode and cathode lead connections, respectively. 100% H_2_ was used as fuel. The fuel and air stream flow rates were controlled separately using mass flow controllers. Cell testing was performed at 750 °C. During the operation, a 400 L/min air flow rate and a 400 mL/min fuel flow rate were used. All samples were loaded at a constant current of 0.3 A/cm^2^ for desired periods. The cell performance and impedance spectra was examined using a potentiostat/galcanostat (Solartron 1470E) equipped with a frequency response analyzer (Solartron 1455A). All data reported in Table 1 were taken after operation for 48 hours for comparison. The impedance spectra and resistance (*R*_s_ and *R*_p_) presented are the ones measured under the DC bias current of 0.3 A/cm^2^. On a Nyquist plot, *R*_s_ is determined by the intercept at the higher frequency end and *R*_p_ is determined by the distance between two intercepts.

ALD coated cells were sectioned and subjected to nanostructural and crystallographic examination using high resolution (HR) Transmission Electron Microscopy (TEM). All the TEM examinations were conducted in the cathode active layer. TEM samples were prepared by mechanical polishing and ion milling in a liquid-nitrogen cooled holder. Electron diffraction, diffraction contrast and HRTEM imaging were performed using a JEM-2100 operated at 200 kV. Chemical analysis was carried out under TEM using energy dispersive X-ray Spectroscopy (EDS).

## Additional Information

**How to cite this article**: Chen, Y. *et al*. Nanoionics and Nanocatalysts: Conformal Mesoporous Surface Scaffold for Cathode of Solid Oxide Fuel Cells. *Sci. Rep*. **6**, 32997; doi: 10.1038/srep32997 (2016).

## Supplementary Material

Supplementary Information

## Figures and Tables

**Table 1:** Impedance data and power densities for the cells with cathodes having different surface architecture.

Cell	Backbone surface architecture	*R*_total_ [Ω/cm^2^]	*R*_s_ [Ω/cm^2^]	*R*_p_ [Ω/cm^2^]	*i* at 0.8 V [A/cm^2^]	*P* at 0.8 V [W/cm^2^]	Factor of enhancement vs baseline
#1	Baseline	0.761	0.235	0.526	0.315	0.252	1
#2	20 nm ZrOx	0.681	0.274	0.407	0.375	0.3	1.2
#3	40 nm ZrOx	0.55	0.185	0.365	0.47	0.376	1.5
#4	60 nm ZrOx	0.569	0.118	0.451	0.351	0.270	1.1
#5	Nano Pt	0.618	0.245	0.373	0.433	0.346	1.4
#6	Nano Pt + 40 nm ZrOx	0.586	0.239	0.347	0.503	0.402	1.6
#7	40 nm ZrOx + nano Pt	0.523	0.250	0.273	0.522	0.418	1.7
