# Supplementary material for: Nanoionics and Nanocatalysts: Conformal Mesoporous Surface Scaffold for Cathode of Solid Oxide Fuel Cells
Source: Sci Rep. 2016 Sep 8;6:32997. doi: 10.1038/srep32997 (PMC5015090; doi:10.1038/srep32997)
Supplement: Supplementary Information [file srep32997-s1.doc]

# Supplementary Information for

# Nanoionics and Nanocatalysts: Conformal Mesoporous Surface Scaffold for Cathode of Solid Oxide Fuel Cells

Yun Chen, Kirk Gerdes, Xueyan Song*


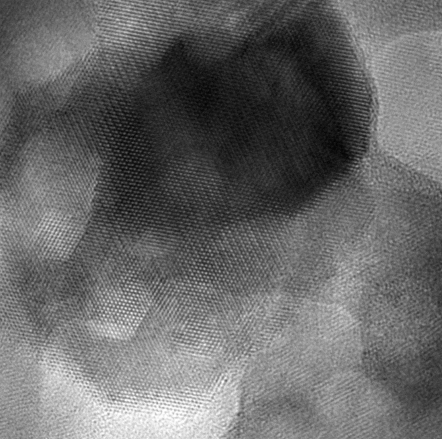


5 nm

[110]

Supplementary **Figure 1.** The high resolution TEM image from [110] zone axis of ZrO2 grains and the corresponding Fast Fourier Transformation showing the ZrO2 possessing tetragonal structure.

a.
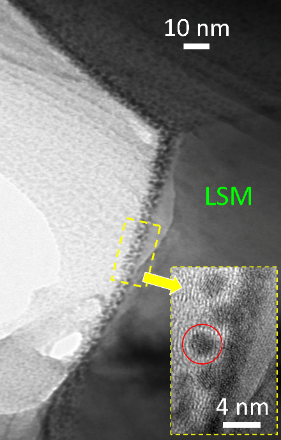
 b.
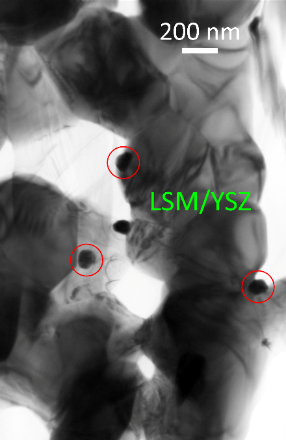


Supplementary **Figure 2.** As-deposited Pt surface layer and the coarsened Pt particles after operation for cell #5. **a**, TEM image shows the uniform Pt surface layer ALD-deposited on the LSM backbone. The insert enlarged image depicts that the Pt layer is composed of ~3 nm discrete Pt particles. **b**, After operation for 100 h, Pt particles evolved to become discrete ~100 nm Pt particles.


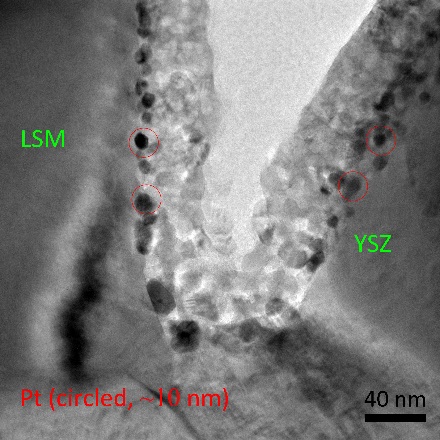


Supplementary **Figure 3.** A two-phase coating of superjacent ZrO_2_ layer subjacent Pt (cell#5). Layered ZrO_2_ architecture contains meso-pores that preserve the gas pathway and disrupt agglomeration of the discrete Pt particles of ~10 nm that were fully pinned to the backbone surface.


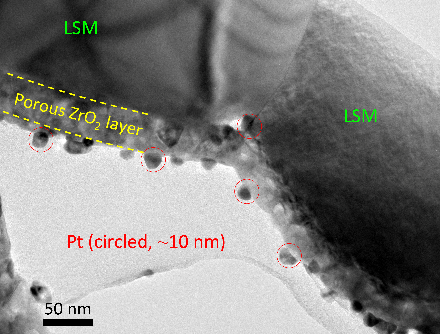


Supplementary **Figure 4.** Morphology of the ZrO_2_/Pt coating layer in Figure 5 (cell #6) after heat treatment at 750°C, presenting disrupt agglomeration of the discrete Pt particles of ~10 nm.
